# Optimal Design of Formulas for a Single Degree of Freedom Tuned Mass Damper Parameter Using a Genetic Algorithm and H_2_ Norm

**DOI:** 10.3390/biomimetics9080450

**Published:** 2024-07-24

**Authors:** Seunggoo Kim, Donwoo Lee, Seungjae Lee

**Affiliations:** School of Industrial Design & Architectural Engineering, Korea University of Technology & Education, 1600 Chungjeol-ro, Byeongcheon-myeon, Cheonan 31253, Republic of Korea; seunggoo@koreatech.ac.kr (S.K.); lov1004ely@koreatech.ac.kr (D.L.)

**Keywords:** tuned mass damper, optimal parameter, dynamic, genetic algorithm

## Abstract

One of the researchers’ concerns in structural engineering is to control the dynamic behavior of structures efficiently. The TMD (tuned mass damper) is one of the effective methods of controlling the vibration of structures, and various numerical techniques have been proposed to find the optimal parameters of the TMD. This paper develops a new explicit formula to derive the optimal parameters of the TMD of a single degree of freedom (SDOF) structure under seismic load using a genetic algorithm (GA). In addition, the state-space model and the H_2_ norm function are used to identify the optimal frequency ratio and damping ratio of the TMD that minimize the overall vibration energy of the structure. The MATLAB curve fitting toolbox is used for the explicit formula proposal, and the validity of the proposed formula is verified through multidimensional performance verification technique. Finally, the TMD parameters of the SDOF structure are applied to the multi-degrees of freedom (MDOF) structure to compare and analyze with the existing research results, and the results of the explicit formula proposed in this paper are confirmed to be excellent. This paper can suggest a new direction for determining the optimal TMD parameters using a GA and can be effectively applied to vibration control problems of various structures.

## 1. Introduction

**Figure 1:**
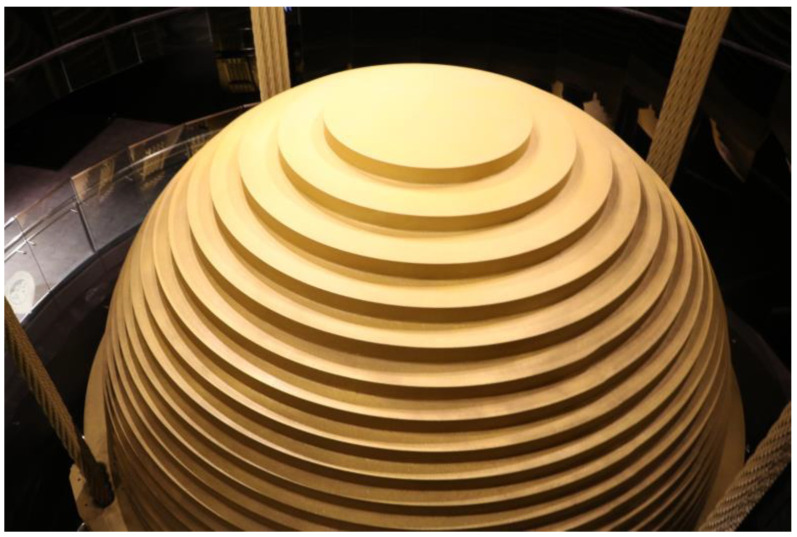
TMD of Taipei 101 in Taiwan.

With the global economy and technological development, structures are becoming increasingly higher and larger. As buildings become taller and larger, controlling the vibrations generated in the structure by horizontal loads such as earthquake or wind loads is one of the critical problems, and in addition to direct vibration control (active, semi-active, passive), various techniques such as structural health monitoring and data recovery have been developed in recent years to improve the efficiency of vibration control [1,2]. In this background, many researchers are interested in TMD (tuned mass damper), a passive control system attached to the main structure because it can effectively control the structure’s dynamic effect [3]. Figure 1 is known as a representative example of TMD, which is applied to the top story of Taipei 101. The TMD applied to Taipei 101 weighs about 660 tons, has a diameter of 5.5 m, and absorbs the structure’s vibration along with eight hydraulic viscous wind dampers.

The TMD began with a proposed anti-rolling tank to reduce the rolling motion and vibration of the hull at sea [4]. This technology can effectively absorb vibrations against external loads at specific frequencies, but the analysis of structures or effective parameters for vibration reduction has yet to be sufficiently performed. Since then, Ormondroid et al. and Den Hartog have introduced a damping system into the TMD to respond efficiently to random vibrations generated by various frequencies [5,6]. They analyzed the mechanism of vibration energy and proposed a new approach using dimensionless variables. However, the damping effect of the main structure needed to be considered, which inevitably caused a difference from the actual phenomenon. Warburton mentioned the importance of TMD parameters and derived optimized parameters of TMD when various external loads were applied to the SDOF (single degree of freedom) structure, considering damping [7].

Previous studies have laid the foundation for effectively reducing the vibration response through TMD optimization. However, it is challenging to derive realistic results in determining the optimal TMD parameter because the existing calculation method provides a limited solution to a complex vibration problem and does not sufficiently reflect the actual conditions, and the results change very sensitively depending on the initial value randomly determined when designing the TMD. An approximate solution called a meta-heuristics algorithm that does not require mathematical differentiation (gradient, degree of inclination) was introduced to improve these shortcomings [3]. A meta-heuristic algorithm performs optimization using natural phenomena as a motif, and various algorithms exist depending on the type of imitated phenomenon. Representative meta-heuristic algorithms include GA (genetic algorithm), PSO (particle swarm optimization), HS (harmony search), ACO (anti-colony optimization), and TLBO (teaching-learning-based optimization). Various researchers have utilized these algorithms to find the optimal parameters for TMD [8,9,10,11,12,13,14,15].

Since meta-heuristic algorithms are time-consuming due to repetitive processes, researchers have sought a systematic and efficient way of designing TMD [16]. As a result, explicit formulas for optimal damping ratio and optimal frequency ratio have been proposed (see Table 1), and Den Hartog first proposed these formulas for SDOF structures [17]. The formulas proposed by Den Hartog influenced Ioi et al. Ioi et al. calculated more accurate optimal damping ratio and optimal frequency ratio by adding the optimal damping ratio of the main structure to the formula proposed by Den Hartog [18]. Warburton et al. proved that if the eigen-frequency is well separated, the parameter optimization of complex systems can be the same as that of SDOF structures [17,18,19,20]. Subsequently, Warburton proposed an explicit formula to obtain the optimal TMD parameters of an SDOF structure subject to white noise random excitation [5]. Warburton’s formula was improved by Bakre et al. and Leung et al. [9,21]. Sadek et al. used curve fitting to derive the formula for the optimal damping ratio and optimal frequency ratio of the TMD affected by the optimal damping ratio of the main structure [22]. In addition, various formulas have been proposed by various researchers to find the optimal TMD parameters [23,24].

Response analysis methods for vibration analysis of structures are classified into time-domain and frequency-domain analysis. Time-domain analysis focuses on how a structure behaves over time under dynamic load conditions and includes information such as displacement, velocity, and acceleration so that the structure’s dynamic behavior can be determined more precisely [15]. Frequency-domain analysis measures the frequency of the dynamic load acting on the structure. It plays an essential role in determining the change and distribution of the energy transferred to the structure. In particular, research has been conducted using the H_2_ norm, which can quantitatively evaluate the overall vibration energy of the main structure over all frequency ranges [8,11,25,26,27]. Recently, attempts have been made to utilize both time-domain analysis and frequency-domain analysis [28]. Although research to determine the optimal parameter of TMD is being conducted in various fields, the setting of TMD parameters may vary depending on various variables, including the main structure. In addition, the parameters proposed by existing studies are limited to specific conditions or structures, making it challenging to apply them to all structures, and a simple and precise formula that can objectively evaluate the efficiency of TMD needs to be proposed. Therefore, this paper proposes a new explicit formula that can derive the optimal parameter of TMD applied to SDOF using a GA among meta-heuristic algorithms.

Section 2 of this paper mentions the necessity of this study by reviewing previous studies, and Section 3 describes the numerical model and numerical method used to propose the optimal model parameters of the TMD. Section 4 proposes an explicit formula for the optimal parameters of the TMD and verifies the validity of the explicit formula using a multidimensional performance verification technique. Section 5 applies the proposed mathematical model to MDOF and compares and reviews it with previous studies. Section 6 derives conclusions.

## 2. Review of Previous Studies

In Section 2, five preceding studies are adopted to analyze the effect on TMD parameter optimization according to various methods of previous studies. To this end, the 10-story structure was taken from the study of Singh et al. to modify the parameters of the main structure [29], and the interpretation methods of each preceding study are summarized in Table 2. Among the previous studies adopted, three are in the frequency domain, one is in the time domain and frequency domain, and one is in the time domain. Each study was optimized using meta-heuristic algorithms such as GA, GSS (golden-section search), DE (differential evolution), CSS (charge system search), and MODS (multi-objective cuckoo search). In addition, LB and UB mean lower boundary and upper boundary. The mass of each story of the main structure is 360 tons, the damping and stiffness of each story are 6200 kN s/m and 650,000 kN/m, and the TMD mass is 108 tons. In addition, an El Centro (1940) N-S seismic wave was used to derive the optimal design parameters.

**Figure 2:**
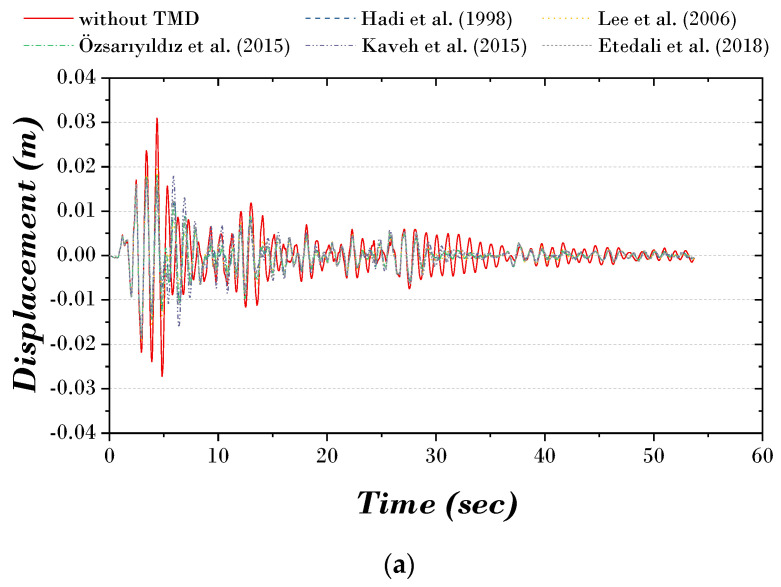

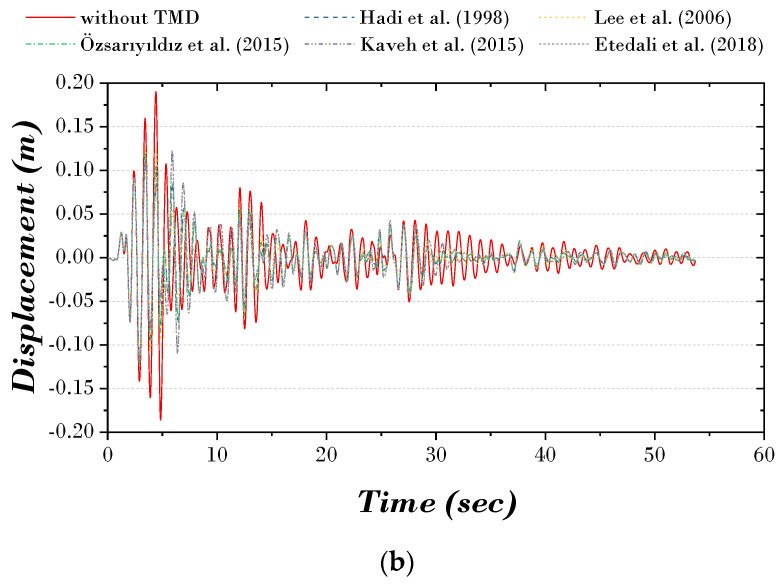
Time history curve of previous studies: (**a**) first story; (**b**) top story [8,11,15,26,28].

**Figure 3:**
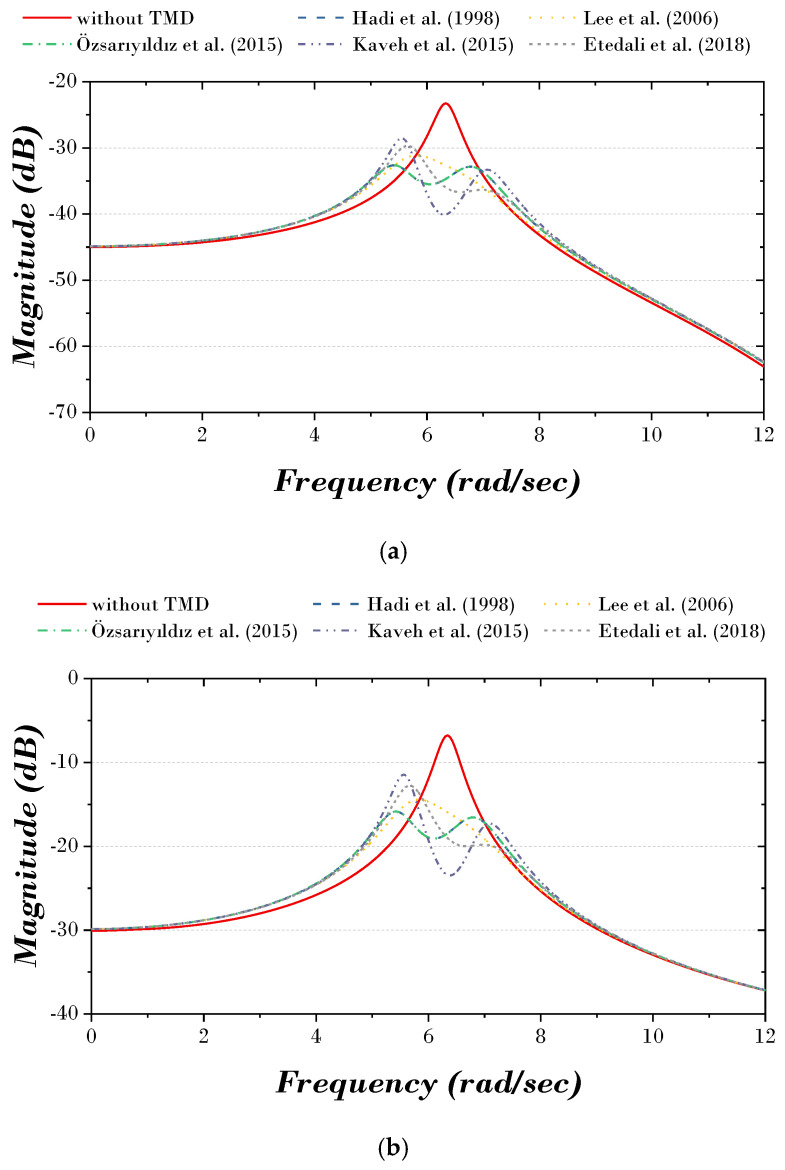
TF curve of previous studies: (**a**) first story; (**b**) top story [8,11,15,26,28].

Figure 2 and Figure 3 show the time history and TF (transfer function) curves of each previous study. The results of all previous studies show that the structure’s displacement and vibration response decrease when the TMD is installed compared to when it is not. Without TMD, a displacement of 0.031 m occurs on the first story and 0.188 m on the top story. After the TMD is installed, the displacement of the top story decreases to 0.122–0.126 m.

**Figure 4:**
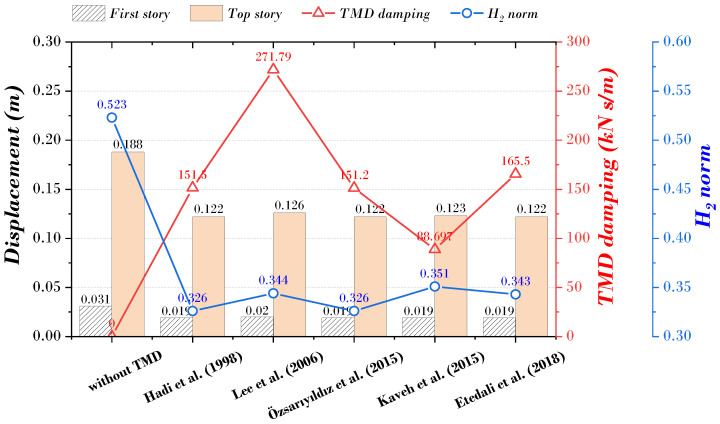
Summary of previous results [8,11,15,26,28].

Figure 4 shows each previous study’s maximum displacement (first, top story), TMD damping, and H_2_ norm. The bar graph represents the displacement, the blue dot represents the TMD damping, and the red dot represents the H_2_ norm. In the studies of Hadi et al., Özsarıyıldız et al., and Etedali et al., the displacement of the top story of the main structure was the smallest at 0.122 m, and TMD damping was the smallest at 88.697 kN s/m in the study of Kaveh et al. The H_2_ norm showed the lowest value at 0.326 in Hadi et al., showing that it is most effective for vibration control. These results indicate that the displacement of the top story is not necessarily the smallest when the TMD damping is the minimum, and there is no direct relationship between the displacement response of the main structure and the TMD damping. This means that various variables, such as the dynamic characteristics of the structure, the mass and stiffness of the TMD parameter, and the characteristics of the load, act in combination, and it is essential to understand the effect of these parameters on the vibration control of the structure from various aspects. In addition, as the value of the H_2_ norm decreases, the displacement response (first, top story) of the main structure tends to decrease, which tends to be more precise than the TMD damping coefficient. This volatility is not sufficient for the methodology proposed in previous studies to be used as a standardized parameter suitable for all situations.

Therefore, this study aims to find a way to precisely evaluate the main structure’s dynamic performance according to the TMD installation using the H_2_ norm and optimize the TMD parameters.

## 3. Numerical Modeling

### 3.1. Equation of Motion

**Figure 5:**
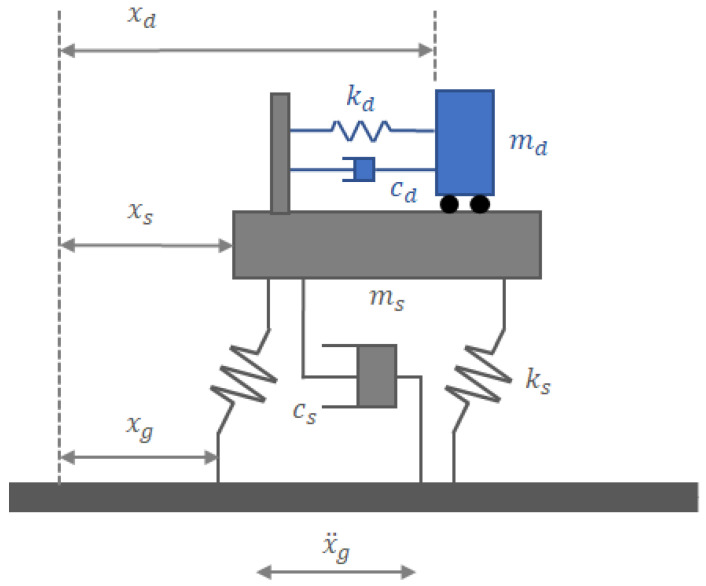
SDOF structure with TMD on top story.

In general, TMD can be easily installed anywhere in the main structure. However, since the maximum amplitude of the mode shape occurs in the top story of the first vibration mode, the optimal location is the top story of the structure [3]. Figure 5 shows a damping SDOF structure. If the difference between the displacement ($x_{s}(t)$) occurring in the structure and the ground displacement ($x_{g}(t)$) is expressed as relative displacement (u(t)), the equation of motion by u(t) can be expressed as Equation (1). Here, M is the mass of the main structure, C is the damping coefficient, and K is the stiffness.

(1) $$M\ddot{u}\left(t\right)+C\dot{u}\left(t\right)+Ku\left(t\right)=-M\left\{1\right\}\ddot{x_{g}}(t)$$

If both sides of Equation (1) are divided by M and organized using C=2ξωM, $K=\omega^{2}M$, it may be expressed as Equation (2). Also, if Equation (2) is converted into a state-space equation, it may be expressed as Equations (3) and (4) [30]. At this time, the size of the state-space equation matrix is twice the size of the motion equation matrix [8].

(2) $$\ddot{u}\left(t\right)+2\xi \omega \dot{u}\left(t\right)+\omega^{2}u\left(t\right)=-\left\{1\right\}\ddot{x_{g}}(t)$$

(3) $$\dot{X}\left(t\right)=AX\left(t\right)+BF(t)$$

(4) $$Y\left(t\right)=R_{\omega }X\left(t\right)+QF(t)$$

where

$X\left(t\right)=\left\{\begin{array}{c} u(t)\\ \dot{u}(t) \end{array}\right\}$

$A=\left[\begin{array}{cc} 0 & I\\ -M^{-1}K & -M^{-1}C \end{array}\right]$

$B=\left[\begin{array}{c} 0\\ M^{-1} \end{array}\right]$

The system’s output matrix depends on the type of output controlled. In this paper, displacement and acceleration are used as controlled outputs. If the controlled output is considered the relative displacement between the story and the TMD, the matrices $R_{\omega }$ and Q can be defined as Equations (5) and (6) [28].

(5) $$R_{\omega }=\left[I_{1\times 1}\;\;0_{1\times 3}\right]$$

(6) $$Q=\left[0_{1\times 1}\right]$$

In addition, the next goal is to find the optimal value of the TMD parameter associated with the system matrix (A).

### 3.2. H_2_ Norm

H_2_ norm has been used in the vibration analysis of structures by external excitation such as wind or seismic loads [8,31]. Considering that the seismic load is a random input, the root mean square (RMS) value, H_2_ norm, is more suitable for evaluating the system’s performance [32].

This paper uses the objective function H_2_ norm in the state-space equation to optimize the TMD parameters. Equation (7) can be derived by converting the state-space equation into TF and substituting and organizing s=iω.

(7) $$H\left(i\omega \right)=\frac{Y\left(i\omega \right)}{F\left(i\omega \right)}=R_{\omega }{\left(i\omega I-A\right)}^{-1}B+Q$$

In Equation (7), the TF is defined as a Laplace transform ratio of the controlled output and a Laplace transform ratio of the input function (external force). ${\left\|H\right\|}_{H_{2}}$ is defined as Equation (8).

(8) $${\left\|H\right\|}_{H_{2}}^{2}=\frac{1}{2\pi }\int_{-\infty }^{+\infty }{\left|H_{x}\left(i\omega \right)\right|}^{2}d\omega =\frac{1}{2\pi }\int_{-\infty }^{+\infty }tr\left[{H_{x}\left(i\omega \right)}^{*}H_{x}\left(i\omega \right)\right]d\omega$$

This paper aims to find the optimal parameters of the TMD that minimize the H_2_ norm of the main structure in all frequency bands when a random external load is applied to it. For this purpose, the GA, one of the meta-heuristic algorithms, is used, and the following optimization problem is defined for the GA:

Find: $\xi_{d,opt}$, $f_{opt}$

(9) $$\mathrm{Minimize}:\;{\left\|H\right\|}_{H_{2}}^{2}$$

Subject to: $\left\{\begin{array}{c} 0.01\leq \xi_{d}\leq 0.3\\ 0.8T_{s}\leq T_{d}\leq 1.5T_{s} \end{array}\right.$

### 3.3. Genetic Algorithm

The meta-heuristic algorithm finds the optimal value of an objective function by imitating natural phenomena. Mathematical techniques using gradient information may fall into local minima. However, the meta-heuristic algorithm is effective in global optimization problems because it probabilistically uses the exploitation and exploration performance [33]. The most representative GA of meta-heuristic algorithms is an algorithm proposed by Holland in 1975 to solve complex optimization problems by mimicking the evolution of living things. The GA maintains diversity by generating new solutions through crossover and mutation operations. This process allows extensive exploration of multiple solutions simultaneously, increasing the likelihood of finding a global optimum [34]. The GA applies to problems that are not mathematically clearly defined, and it is also successfully used in structural engineering to find the minimum weight of structures.

In order to maximize the efficiency of GA, the following improved GA are applied in this study: first, to overcome the limitations of the binary number coding method of traditional GA, a real number coding method is adopted to overcome the limitations of the binary number coding method of traditional GA. The binary number coding method facilitates genetic computation, but the search space is enlarged, which may slow down the computation speed. The real number coding method is advantageous because it can efficiently represent a wider range of values and accurately reflect the continuity between genes [35]. Second, to improve the efficiency of the genetic algorithm, we set the parameters of the population size, crossover operation, and mutation operation on a ratio basis. This keeps the proportion of individuals involved in the computation constant even as the population size changes, allowing it to respond flexibly to different population sizes and increasing the probability of reaching the global optimum. This allows it to respond efficiently when the environment or problem conditions change dynamically. Third, the selection process of solutions in GA has been done through elitist strategies and the roulette wheel method [36].

The basic procedure of GA is to set parameters, generate initial values, evaluate fitness, select a solution, perform crossover operations, perform mutation operations, and evaluate fitness again. The parameters of GA used in this paper are set based on the research results of Suryadi et al. [37], and the population size, number of generations, crossover and mutation rates, elitist strategy, and iteration termination criteria are as shown in Table 3.

Algorithm 1 is the pseudo code of the GA.
**Algorithm 1.** The Psuedo code of the GA**Begin**      t = 0      Initialize P(t)       Evaluate P(t)      Select P(t) from P(t) using elite and roulette wheel      **while** (not termination condition) **do**            crossover P(t + 1) to yield C(t)            mutate C(t)            evaluate C(t)            select P(t + 1) from P(t) and C(t)             t = t + 1      **end** **end**

## 4. TMD Optimization for Damped SDOF Structure

### 4.1. Extraction of TMD Parameters

According to the analysis procedure using GA, the period and damping ratio of the TMD are used as design variables, and the parameters of the TMD are optimized to minimize the H_2_ norm of the main structure when receiving the input of ground acceleration across all frequencies. In the parameter optimization process of TMD, the damping ratio and frequency ratio are considered essential parameters, and an appropriate mass ratio is assumed in advance. This is because an unrealistically high value is obtained if the mass ratio is considered a direct design variable in the optimization process [38].

Table 4 is the design variable used for the TMD parameter operation in this paper. According to the parameter conditions of the TMD, a total of 28 mass ratios ranging from 1% to 10% of the total mass of the structure were adopted, and the optimum damping ratio and optimum frequency ratio were extracted for a total of 168 cases (see Table A1) by adopting the damping ratio (1, 2, 3, 5, 7.5, 10%) commonly used in construction and civil engineering structures. It can represent the various dynamic behaviors of the structure and is intended to cover the range observed in various types of structures under general conditions. The cycle of the TMD was explored between 0.8 and 1.5 times the intrinsic cycle of the structure. At this time, $T_{s}=1$ is to simplify the mathematical expression and make the result dimensionless to extract the general formula of the TMD parameter so that it can be applied to various systems. After obtaining the generalized formula, the actual cycle or frequency of the target structure can be reintroduced into the equation to apply it to a specific structure.

**Figure 6:**
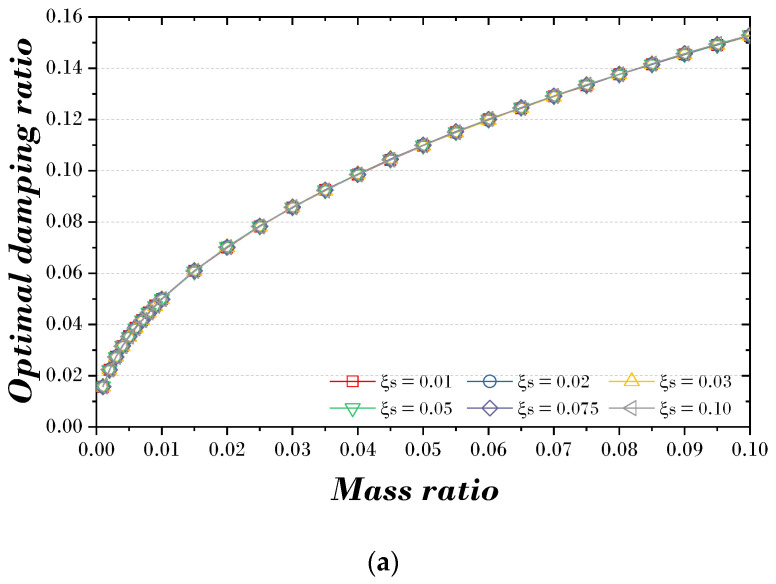

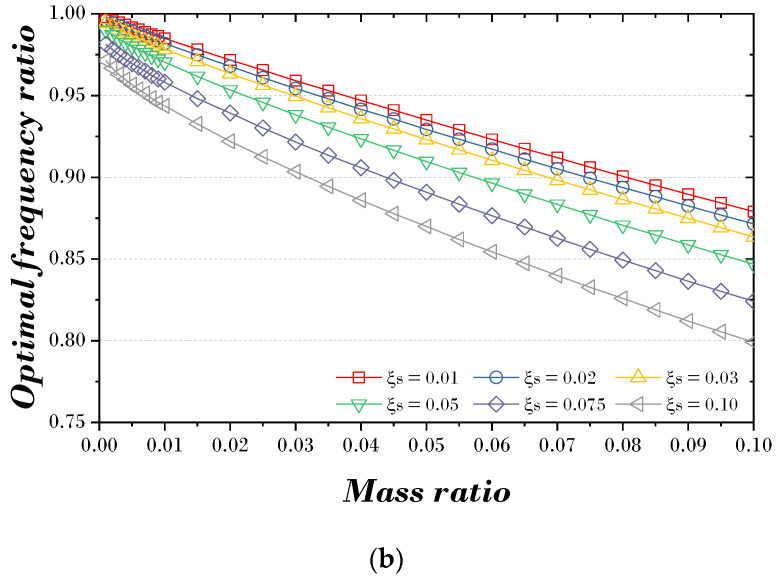
Optimal parameters curve-fitting of TMD: (**a**) optimal damping ratio; (**b**) optimal frequency ratio.

Figure 6 is a two-dimensional curve-fitting graph of the optimal damping ratio and optimal frequency ratio of the TMD according to the mass ratio and the damping ratio of the main structure when external excitation acts on the damping SDOF structure. As shown in Figure 6a, the optimal damping ratio of the TMD is not significantly affected by the main structure’s damping ratio. As the mass ratio increases, the optimal damping ratio of the TMD also increases nonlinearly. On the other hand, as shown in Figure 6b, the optimal frequency ratio of the TMD is greatly affected by the main structure’s damping ratio. As the mass ratio increases, the optimal frequency ratio of the TMD decreases nonlinearly.

### 4.2. Deriving Explicit Formulas for TMD Parameters

Deriving an accurate mathematical formula for optimal parameters is a very difficult task, considering the complexity and minimization conditions. To this end, curve fitting using multi-variable regression analysis was applied, and the mathematical relationship between independent and dependent variables was quantified. The model that best fits the given data was selected by evaluating the suitability of various functional forms. By minimizing errors and by optimizing the parameters for the selected model, two explicit formulas for the optimal parameters of the TMD system were finally derived.

Sadek et al. considered the natural frequency ($w_{s}$), damping ratio ($\xi_{s}$), mass ratio (μ), frequency ratio (f), and TMD damping ratio ($\xi_{d}$) of the structure to derive the explicit formula for a damping SDOF structure with TMD. It was changed to the system matrix A of the state-space equation, and an explicit formula for estimating the optimal parameter of TMD was proposed using curve fitting after eigenvalue analysis for matrix A. The explicit formula proposed by Sadek et al. is shown in Equations (10) and (11) [22].

(10) $$\xi_{d,opt}=\frac{\xi_{s}}{1+\mu }+\sqrt{\frac{\mu }{1+\mu }}$$

(11) $$f_{opt}=\frac{1}{1+\mu }\left[1-\xi_{s}\sqrt{\frac{\mu }{1+\mu }}\right]$$

In this study, based on the research of Sadek et al., the mass ratio and the damping ratio of the main structure were used as the main parameters. Curve fitting was used to find the optimal parameter, and through this, an explicit formula for TMD parameter optimization in the nonlinear model was developed. In this process, two new approaches to TMD parameter estimation for the nonlinear model were presented.

First, the nonlinear model was assumed, as shown in Equations (12) and (13). The parameters used the MATLAB R2023a curve fitting toolbox and were used in the simulation for the explicit formulation of TMD parameters. The result values of each parameter derived using curve fitting are shown in Table 5, and Explicit Formula 1 proposed is shown in Equations (14) and (15).

(12) $$\xi_{d,opt}=\frac{a\times \xi_{s}}{1+b\times \mu }+\sqrt{\frac{c\times \mu }{1+d\times \mu }}$$

(13) $$f_{opt}=\frac{1}{1+a^{\prime }\times \mu }\left[1-b^{\prime }\times \xi_{s}\sqrt{\frac{c^{\prime }\times \mu }{1+d^{\prime }\times \mu }}\right]$$

(14) $$\xi_{d,opt}^{1st}=\frac{0.0031\xi_{s}}{1+9.7353\mu }+\sqrt{\frac{0.2484\mu }{1+0.6354\mu }}$$

(15) $$f_{opt}^{1st}=\frac{1}{1+1.2263\mu }\left[1-3.8063\xi_{s}\sqrt{\frac{1.0708\mu }{1+5.2784\mu }}\right]$$

Second, Equations (10) and (11), proposed by Sadek et al. [22], contain the same $\sqrt{\mu /1+\mu }$. From the first result, it can be seen that the damping ratio coefficient (*a*) of the main structure in the molecule of the optimal damping ratio is 0.0031, which is a minimal value. In addition, as shown in Figure 6a, the optimal damping ratio of the TMD is not significantly affected by the damping ratio of the main structure. Therefore, a nonlinear model was assumed, as shown in Equations (16) and (17). Table 6 shows the parameter result values of the nonlinear model using curve fitting. However, for convenience in the calculation, the parameter results of $f_{opt}$ were rounded and expressed as the first digit of the decimal point, and the proposed Explicit formula 2 can be expressed as Equations (18) and (19).

(16) $$\xi_{d,opt}=\sqrt{\frac{a\times \mu }{1+b\times \mu }}$$

(17) $$f_{opt}=\frac{1}{1+a^{\prime }\times \mu }\left[1-b^{\prime }\times \xi_{s}\sqrt{\frac{a\times \mu }{1+b\times \mu }}\right]$$

(18) $$\xi_{d,opt}^{2nd}=\sqrt{\frac{\frac{\mu }{4}}{1+\frac{2}{3}\mu }}$$

(19) $$f_{opt}^{2nd}=\frac{1}{1+1.2\mu }\left(1-7.0\xi_{s}\xi_{d,opt}^{2nd}\right)$$

### 4.3. Verification of Explicit Formulas for Parameter of TMD

For parameter optimization of TMD, the two explicit formulas proposed in this paper and 168 parameter data derived through GA are compared and analyzed. This determines the optimal damping ratio and optimal frequency ratio, and the validity of the explicit formula proposed in this paper is verified using multidimensional performance verification techniques such as scatterplot and correlation coefficients, error and fitting error, MAE (mean absolute error), and (NRMSE) normalized root mean square error.

**Figure 7:**
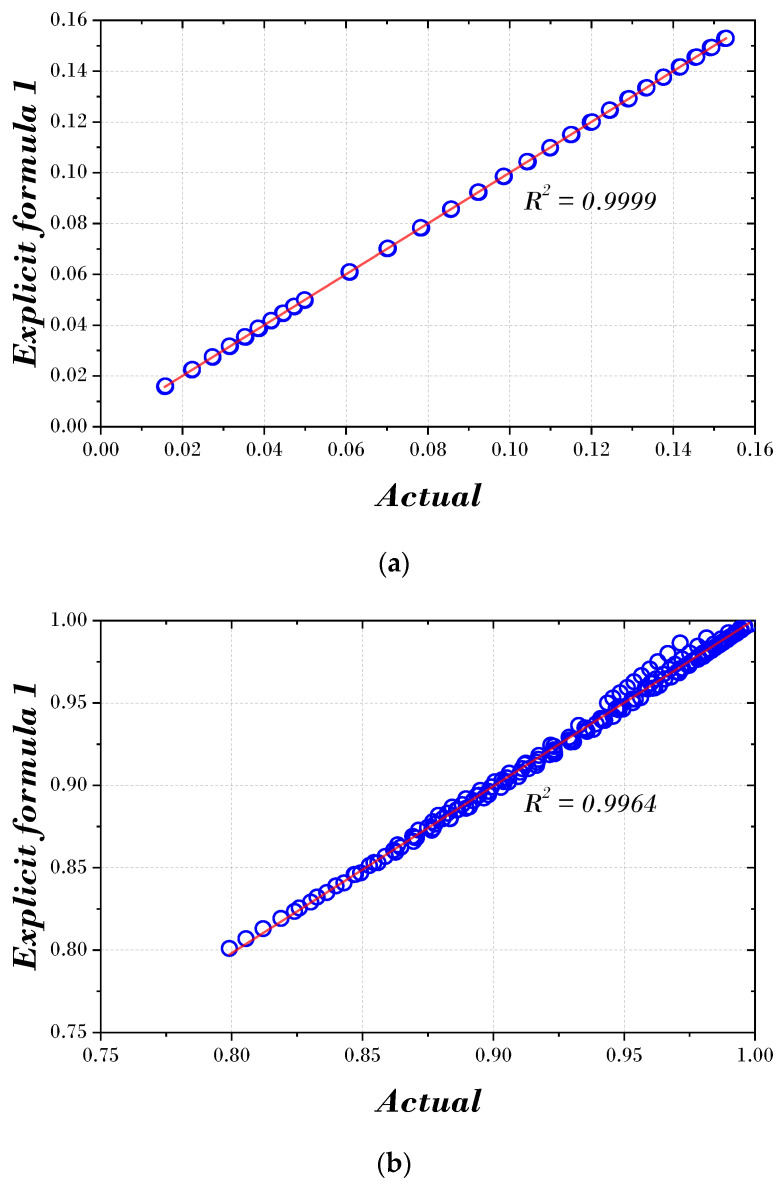
Scatter and correlation plot using Explicit formula 1: (**a**) optimal damping ratio; (**b**) optimal frequency ratio.

**Figure 8:**
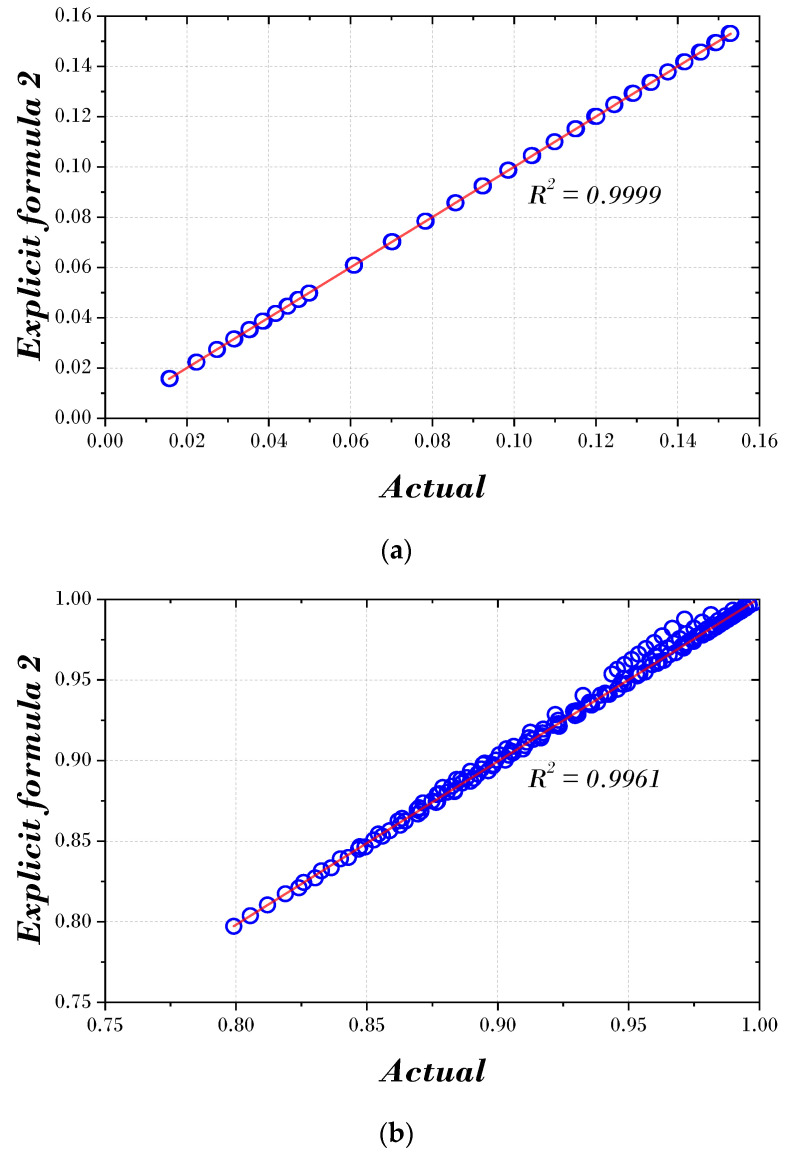
Scatter and correlation plot using Explicit formula 2: (**a**) optimal damping ratio; (**b**) optimal frequency ratio.

Figure 7 and Figure 8 express the actual value and the results using the two explicit formulas proposed in this paper. In both figures, the optimal damping and frequency ratios are concentrated around the regression line, and the data points are closely aligned along the line. In addition, the correlation ($R^{2}$) of the optimal damping ratio is 0.9999, and the correlation ($R^{2}$) of the optimal frequency ratio is 0.9964 or 0.9961, indicating a robust correlation.

**Figure 9:**
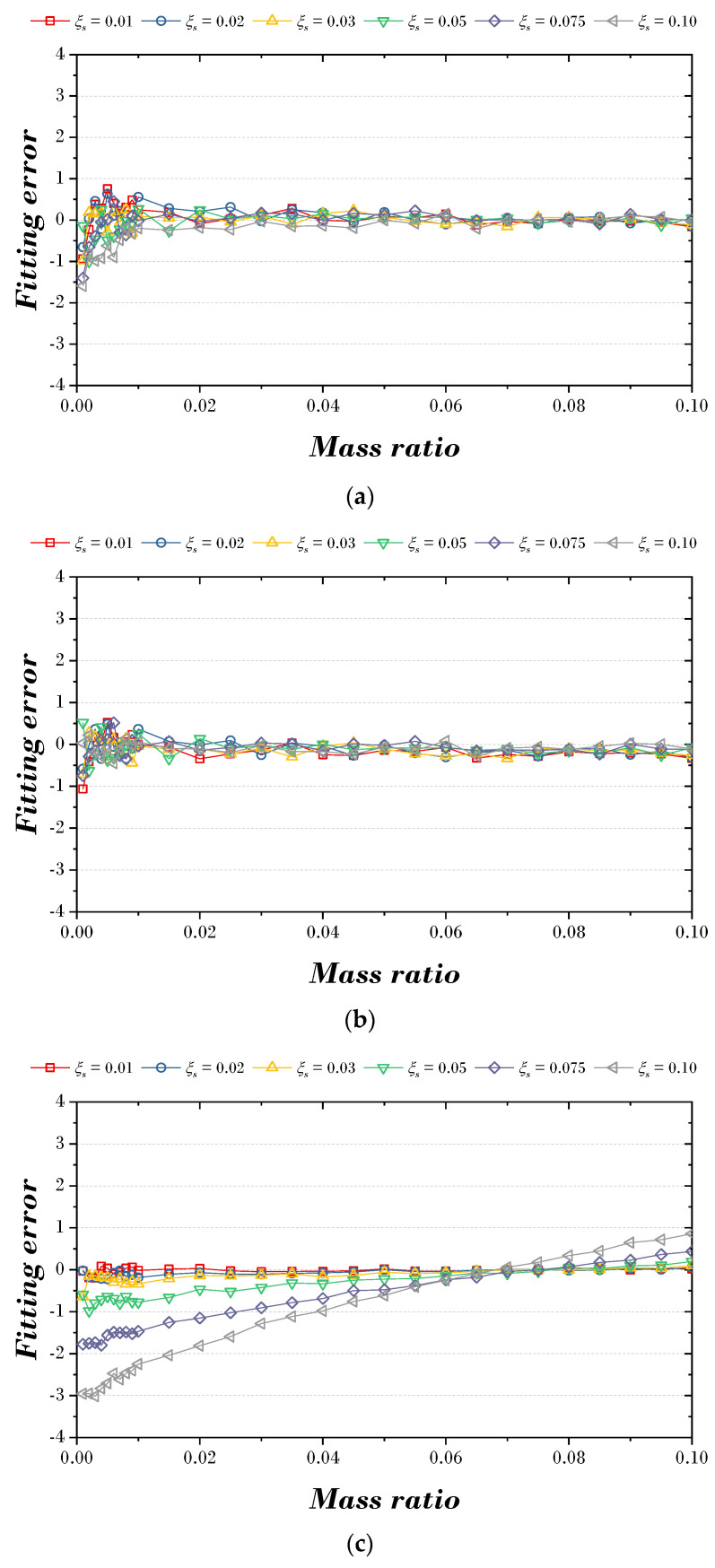
Fitting error between proposed explicit formula and optimal damping ratio: (**a**) Explicit formula 1; (**b**) Explicit formula 2; (**c**) Leung et al [9].

**Figure 10:**
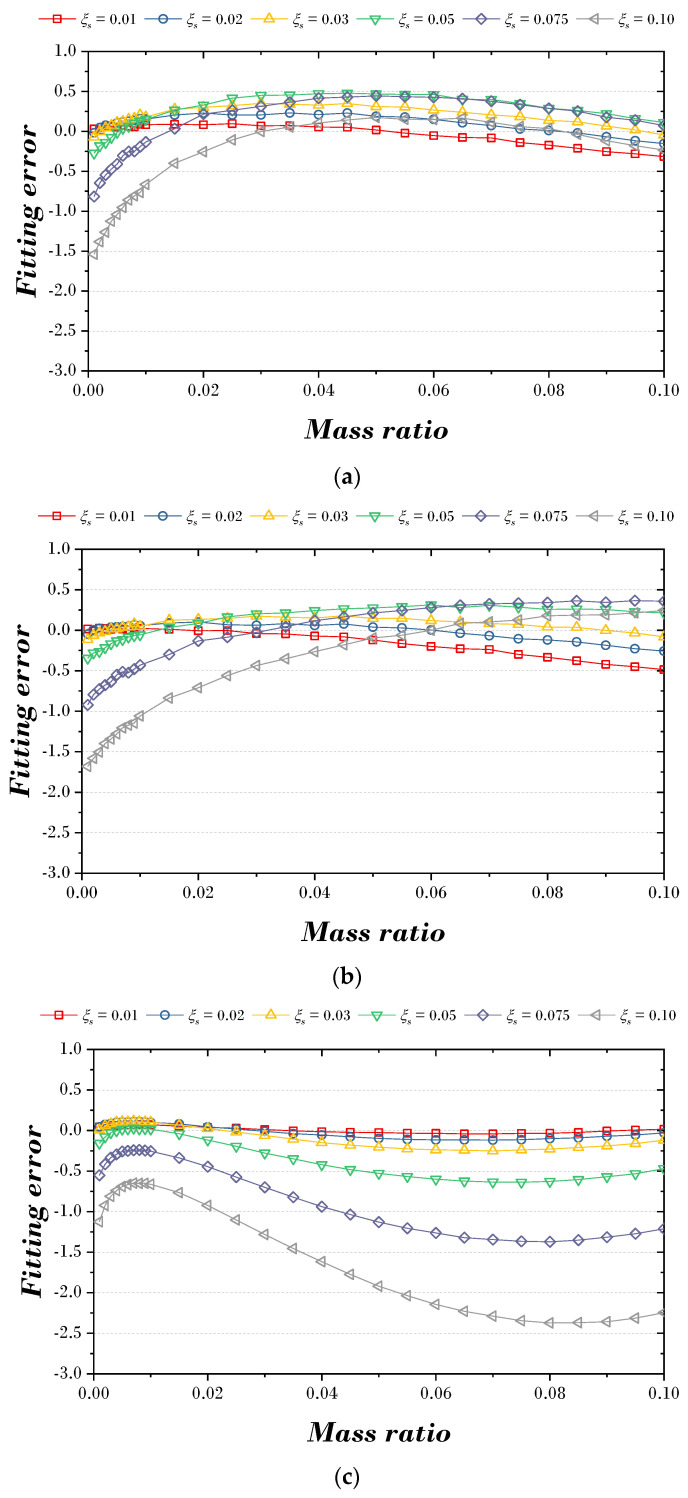
Fitting error between proposed explicit formula and optimal frequency ratio: (**a**) Explicit formula 1; (**b**) Explicit formula 2; (**c**) Leung et al [9].

Figure 9 and Figure 10 are the figures that express the fitting error according to the explicit formula proposed in this paper and the optimal parameters of the previous study [9]. Figure 9a,b shows the fitting error results for two explicit formulas for the optimal damping ratio. When the mass ratio is less than 0.01, the fitting error tends to range between −1.0 and 1.0, whereas when the mass ratio exceeds 0.01, the fitting error tends to stabilize between −0.3 and 0.3. However, in the case of Figure 9c, the fitting error tends to increase from side to side based on the mass ratio of 0.07. Figure 10a,b is the fitting error results for two explicit formulas for the optimal frequency ratio. The fitting error appeared to be less than 2.0 at the mass ratio of 0.02, and after that, the fitting error tended to decrease as the mass ratio increased. However, in the case of Figure 10c, the fitting error was about 1.0 at a small mass ratio, but the fitting error tended to increase as the mass ratio increased.

**Figure 11:**
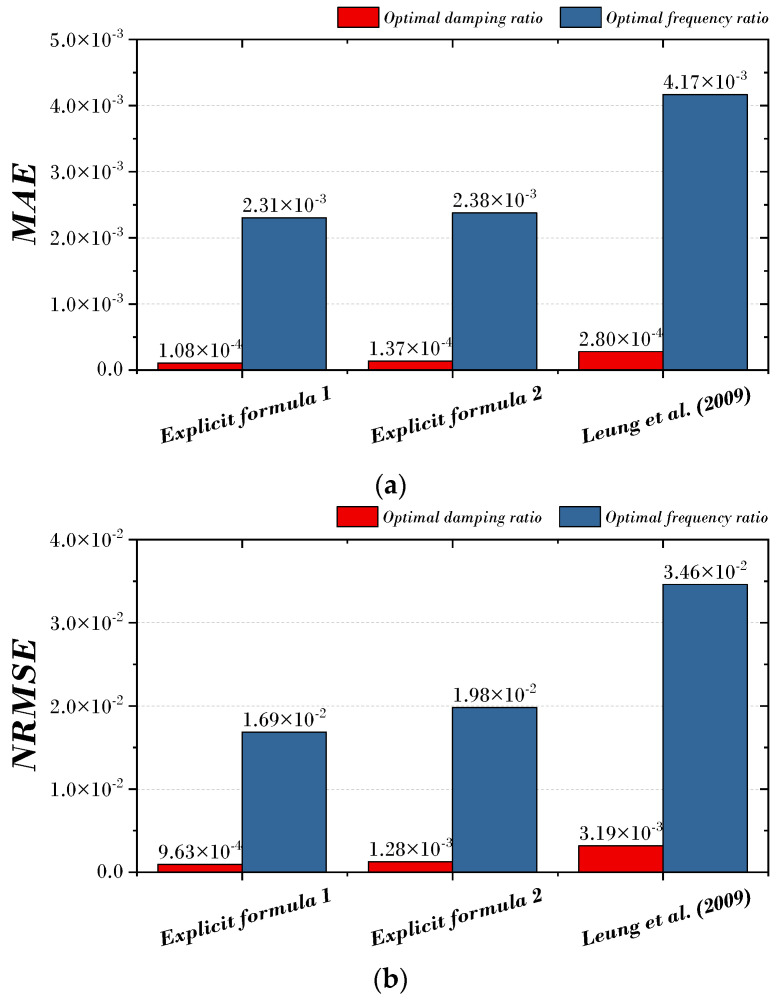
Comparison of the proposed explicit formula and previous study. (**a**) MAE; (**b**) NRMSE [9].

For the comparison of the results of Figure 9 and Figure 10, the MAE (mean absolute error) and NRMSE (normalized root mean squared error) for the optimal damping ratio and the optimal frequency ratio are shown in Figure 11. In Figure 11a, MAE was derived as 1.08 × 10^−4^ and 2.31 × 10^−3^ in Explicit formula 1, 1.37 × 10^−4^ and 2.38 × 10^−3^ in Explicit formula 2, and 2.80 × 10^−4^ and 4.17 × 10^−3^ in previous studies. In Figure 11a, NRMSE was derived as 9.63 × 10^−4^ and 1.69 × 10^−2^ in Explicit formula 1, 1.28 × 10^−4^ and 1.98 × 10^−2^ in Explicit formula 2, and previous studies as 3.19 × 10^−3^ and 3.46 × 10^−2^.

Based on these results, it can be confirmed that both explicit formulas proposed in this paper match the optimal parameters of the TMD better than previous studies.

## 5. Optimal TMD Design for Damped MDOF Structure

The explicit formulation of the optimal parameters of the TMD presented in Section 4 was used to perform the optimal design for two cases of MDOF (10-story) structure in Section 5. First, the results of TMD parameters and main structural responses derived from the explicit formulation proposed in previous studies [9,21,22,24] of Table 1 and the explicit formulation proposed in this study are compared. Second, the results of TMD parameters and main structural responses derived by various numerical exploration techniques, including meta-heuristic algorithms in previous studies [8,15,22,28] were compared with those derived by Explicit formulas 1 and 2 proposed in this study.

Sadek et al. improved the method proposed by Villaverde and proposed a method of estimating the optimal parameters of TMD applicable to SDOF and MDOF structures through mathematical modeling and eigenvalue analysis. Table 1 shows the optimal parameter estimation equation of TMD applicable to SDOF structures, and the optimal parameter estimation equation of TMD applicable to MDOF structures is shown in Equations (20) and (21) [22,23].

(20) $$\xi_{d,opt}=\emptyset \left[\frac{\xi_{s}}{1+\mu }+\sqrt{\frac{\mu }{1+\mu }}\right]$$

(21) $$f_{opt}=\frac{1}{1+\emptyset \mu }\left[1-\xi_{s}\sqrt{\frac{\emptyset \mu }{1+\emptyset \mu }}\right]$$

In general, a method of converting an MDOF structure into an SDOF structure for a specific mode (first mode) is used to simplify the MDOF structure. Rana et al. showed that the equation describing the dynamic behavior of the TMD in the MDOF structure may be simplified by normalizing the specific mode shape vector of the structure to the location of the TMD [39]. In this process, the first mode shape ($\emptyset_{1}$) for the unit mode participation coefficient ($\Gamma_{i}$) and mode mass are used, as shown in Equation (22). Here, $M_{i}=\emptyset_{i}^{T}M\emptyset_{i}$. In Equations (22) and (23), $\emptyset_{i}^{T}$ is a transposition of the i-th mode shape, and $M_{i}$ is a mode mass of the i-th mode. Accordingly, complex calculations may be avoided in the TMD optimal design of MDOF structures, and a simple design approach commonly used in SDOF structures may be used instead. Sadek et al., Hadi et al., and Yucel et al. used this method to derive the parameters of TMD for application to the MDOF structure [3,8,22,40].

(22) $$\Gamma_{i}=\frac{\emptyset_{i}^{T}M\left\{1\right\}}{M_{i}}\;$$

(23) $$\mu =\frac{m_{d}}{M_{1}}=\frac{m_{d}}{\emptyset_{1}^{T}\left[M\right]\emptyset_{1}}$$

In this study, the response of the MDOF structure installed with the TMD and the optimal parameters of the TMD were estimated. For the MDOF structure, the model of Sadek et al. was referenced [22], and the structure’s mass, damping, stiffness, and first mode shape are shown in Table 7. In addition, the damping of the structure is assumed to be Rayleigh and can be expressed as C=αK+βM. The damping matrix (C) is proportional to the stiffness and mass matrices, and α and β are constants. Since the MDOF structure of Sadek et al. is related only to the first mode, the damping matrix may be assumed to be proportional to the stiffness or mass matrix. Therefore, damping was derived by considering C=0.0129K, where the damping matrix is proportional to the stiffness matrix [6]. In addition, the mass ratio of the structure is assumed to be 0.05, and since $M_{1}$ is 1109.21 ton, the mass of the TMD is 55.45 ton. The critical damping ratio and the critical frequency of the first mode of the structure are 0.02 and 0.495 Hz, respectively.

### 5.1. Optimal TMD Parameters Using Explicit Formulas

**Figure 12:**
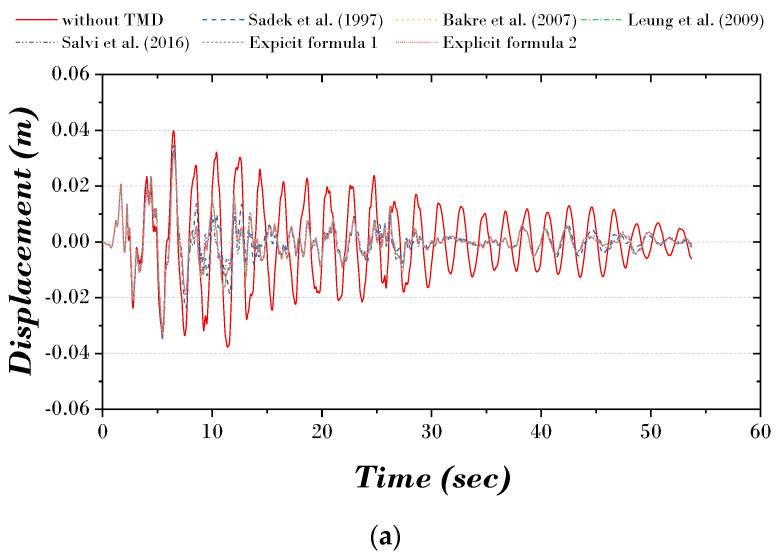

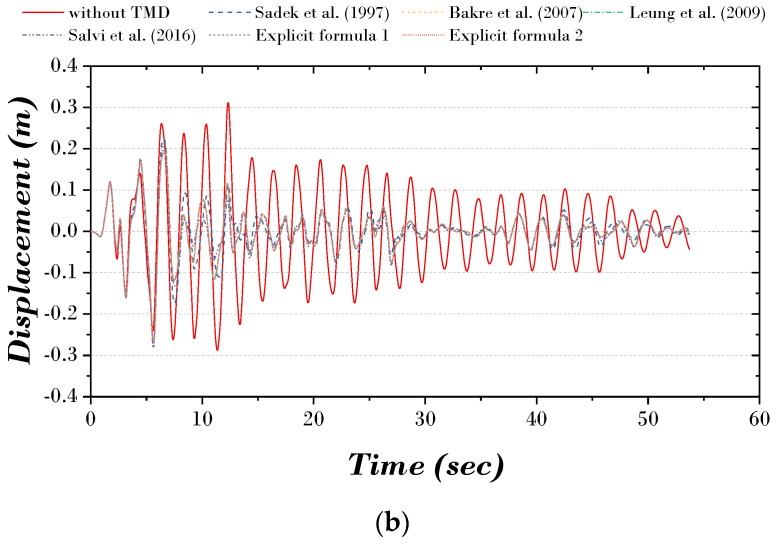
Time history curve of MDOF structure: (**a**) first story; (**b**) top story [9,21,22,24].

**Figure 13:**
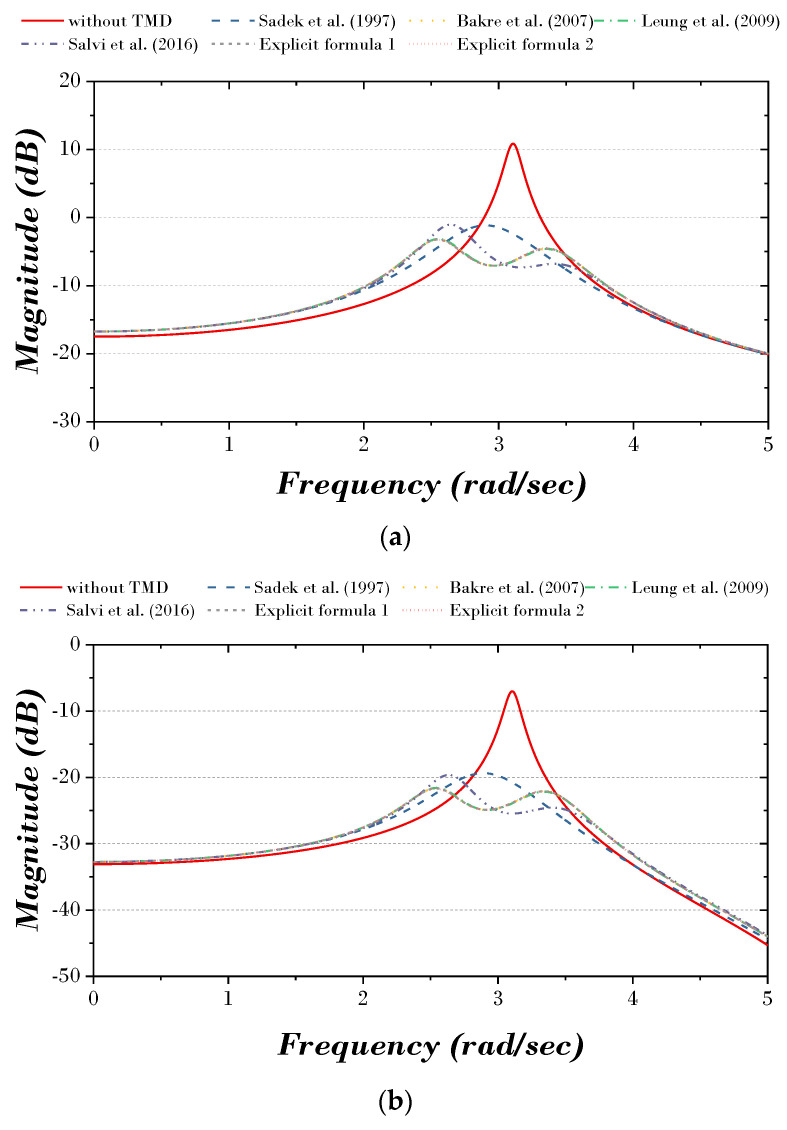
TF curve of MDOF structure: (**a**) first story; (**b**) top story [9,21,22,24].

The TMD parameters, the displacement response of the main structure (first, top story), and the H_2_ norm are derived by the explicit formulation proposed in this paper, and those derived by the explicit formulation of previous studies [9,21,22,24] are shown in Table 8. Based on the results, the time history and TF curves are shown in Figure 12 and Figure 13. It can be seen that with the TMD installed, the displacement and magnitude are reduced in both the first and top story of the MDOF structure compared to without the TMD.

**Figure 14:**
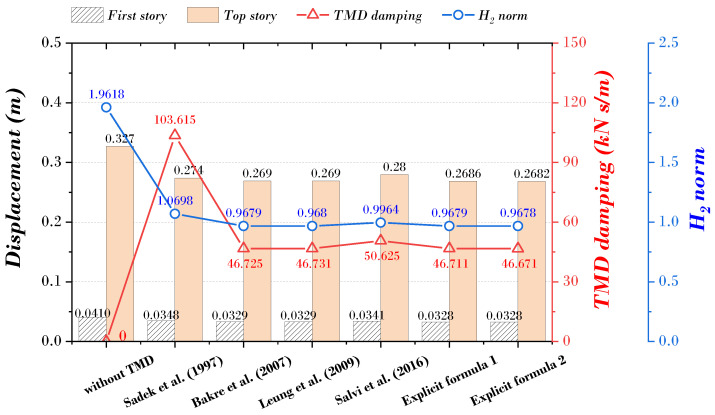
Comparison of displacement (first, top story), TMD damping, and H_2_ norm for MDOF structure derived from explicit formulas [9,21,22,24].

The results of displacement (first, top story), TMD damper, and H_2_ norm derived from all studies are expressed as shown in Figure 14. The top story displacement of the main structure was 0.2686 m (Explicit formula 1) and 0.2682 m (Explicit formula 2), and the result of the explicit formula proposed in this paper was the smallest, decreasing by about 17.86% and 17.98% compared to when the TMD was not installed. The damping coefficient of TMD was also the smallest, with 46.711 kN s/m (Explicit formula 1) and 46.671 kN s/m (Explicit formula 2) derived from the explicit formula proposed in this paper, and decreased by about 14.95% and 14.94% compared to when without TMD. Finally, the H_2_ norm results of the explicit formula proposed in this paper were derived as 0.9679 and 0.9678, reducing about 50.66% and 50.67%, confirming that they most effectively controlled the vibrations generated in the structure.

### 5.2. Optimal TMD Parameters Using Explicit Formulas 1 and 2 and Previous Studies

The TMD parameters and the main structure displacement response, H_2_ norm, obtained using various numerical exploration techniques, including meta-heuristic algorithms, for an example model (Sadek et al. [22]) in previous studies [8,15,22,28] are shown in Table 9, and the results are compared with those obtained using the explicit formulation proposed in this study.

**Figure 15:**
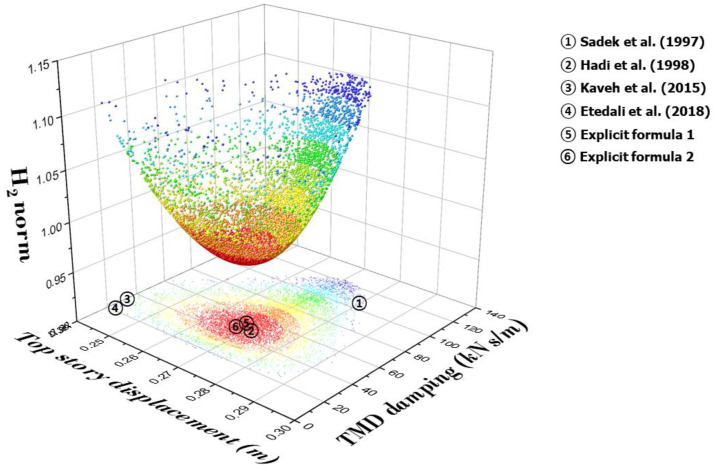
Comparison of top story displacement and TMD damping relative to the H_2_ norm from previous studies [8,15,22,28].

Figure 15 is a three-dimensional graph of the results of 10,000 analyses of an example model (Sadek et al. [22]) using the objective function H_2_ norm and GA. The graph shows the H_2_ norm for the displacement of the top story of the main structure and the TMD damping. The data distribution of the objective function H_2_ norm is a convex function with a minimum value. In Figure 15, each red point represents the optimal solution, and when the results of Table 9 are plotted in Figure 6, it can be seen that the results of Explicit formula 1 and Explicit formula 2 are concentrated in the region of the optimal solution.

This shows that using the H_2_ norm to set the optimal TMD parameters can quantitatively evaluate the dynamic performance of the structure and the effectiveness of the TMD installation and can play an important role in improving the safety and durability of the structure. The explicit formulation based on it enables researchers or engineers to quickly derive the optimal TMD parameters in the TMD design process without the need for complex numerical analysis procedures or simulations.

## 6. Conclusions

As structures become increasingly high-rise and large, controlling the vibration generated by horizontal loads is an important issue. Therefore, this paper proposes a new explicit formula that can identify the problems of previous studies and derive the optimal parameters of the TMD applied to the SDOF structure using GA. The conclusion of the paper is as follows.

- Although the optimal parameters of the TMD were derived through various numerical techniques in previous studies, the setting of the TMD parameters may vary due to the influence of the main structure and various variables. In addition, it was confirmed that the parameters of the TMD proposed by previous studies were insufficient to be applied to all structures, and a similar trend was confirmed in the relationship between the H_2_ norm and displacement response (first, top story).
- Two explicit formulas were derived for optimizing TMD using GA and MATLAB curve fitting toolbox. The MAE and NRMSE for these two formulas’ optimal damping and frequency ratios were compared with previous studies. As a result, they decreased by 61.43% and 44.68%, respectively, in MAE and by 69.77% and 51.32%, respectively, in NRMSE.
- As a result of performing the optimal TMD design installed on the MDOF structure, it was confirmed that the proposed two explicit formulas were most effective in reducing the structural responses, and the result of Explicit formula 2 was better than Explicit formula 1. In addition, the data distribution of the objective function H2 norm is a convex function with a minimum value. The results of Explicit formula 1 and Explicit formula 2 are concentrated in the region of the optimal solution. In this study, two explicit formulas were proposed that can quickly derive the optimal parameters of the TMD without complicated numerical analysis or simulation in the design process. In particular, the accuracy and reliability of the proposed model were secured through the multidimensional performance verification technique, and the basis for quantitatively evaluating the results of applying the optimal parameters of the TMD was laid. Compared to previous studies, the proposed two explicit formulas have superior response performance of the structure and are expected to be essential reference materials for TMD design.

## Figures and Tables

**Table 1:** Explicit formulas for optimal parameters of TMD applied to SDOF structures.

**Index**	Optimal Parameters of TMD	
	$\mathrm{Optimal}\;\mathrm{Damping}\;\mathrm{Ratio}\;(\xi_{d,opt}$)	$\mathrm{Optimal}\;\mathrm{Frequency}\;\mathrm{Ratio}\;(f_{opt}$)
Den Hartog [17]	$\sqrt{3\mu /8\left(1+\mu \right)}$	$1/\left(1+\mu \right)$
Ioi et al. [18]	$\sqrt{3\mu /8\left(1+\mu \right)}+p\left(\mu ,\xi_{s}\right)$	$1/\left(1+\mu \right)-p\left(\mu ,\xi_{s}\right)$
Warburton [7]	$\sqrt{\mu \left(1-\frac{\mu }{4}\right)/4\left(1+\mu \right)\left(1-\frac{\mu }{2}\right)}$	$\sqrt{1-\frac{\mu }{2}}/\left(1+\mu \right)$
Sadek et al. [22]	$\xi_{s}/\left(1+\mu \right)+\sqrt{\mu /\left(1+\mu \right)}$	$\left(1-\xi_{s}\sqrt{\frac{\mu }{1+\mu }}\right)/\left(1+\mu \right)$
Bakre et al. [21]	$\sqrt{\mu \left(1-\frac{\mu }{4}\right)/4\left(1+\mu \right)\left(1-\frac{\mu }{2}\right)}$	$\begin{array}{ll} \sqrt{1-\frac{\mu }{2}}/\left(1+\mu \right) & +\sqrt{\mu }\xi_{s}(-3.79441+9.87259\sqrt{\mu }\\ & -15.2978\mu )\\ & +\sqrt{\mu }\xi_{s}^{2}(-13.6731+19.1284\sqrt{\mu }\\ & +21.7049\mu ) \end{array}$
Leung et al. [9]	$\sqrt{\mu \left(1-\frac{\mu }{4}\right)/4\left(1+\mu \right)\left(1-\frac{\mu }{2}\right)}-5.3024\xi_{s}^{2}\mu$	$\begin{array}{ll} \sqrt{1-\frac{\mu }{2}}/\left(1+\mu \right) & +\sqrt{\mu }\xi_{s}(-4.9453+20.2319\sqrt{\mu }\\ & -37.9419\mu )\\ & +\sqrt{\mu }\xi_{s}^{2}\left(-4.8287+25.0\sqrt{\mu }\right) \end{array}$
Salvi et al. [24]	$\sqrt{\mu }/2$	$1-\sqrt{3\mu }\left(2\mu /3+3\xi_{s}/2\right)$

**Table 2:** Design parameters of the main structure and TMD in previous studies.

Index		Hadi et al. [8]	Lee et al. [26]	Özsarıyıldız et al. [11]	Kaveh et al. [28]	Etedali et al. [15]
Domain		Frequency	Frequency	Frequency	Frequency + Time	Time
Method		GA	GSS	DE	CSS	MOCS
Parameters of main structure	Mass	360 ton				
	Damper	6200 kN s/m				
	Stiffness	650,000 kN/m				
Parameters of TMD	Mass	108 ton (3% of the total mass of the main structure)				
	Damper	LB: 0 kN s/m, UB: 1000 kN s/m				
	Stiffness	LB: 0 kN/m, UB: 4000 kN/m			LB: 0 kN/m, UB: 5000 kN/m	

**Table 3:** The parameters of the GA.

Parameters	Value
population size	30
number of generations	200
crossover and mutation rates	0.5:0.5
elitist strategy	3
iteration termination criteria	5000

**Table 4:** Design variables for TMD parameters analysis.

Parameter	Design Variables
Mass ratio (μ)	$0.1–10\%\;\mathrm{of}\;M_{s}$$\;(A\;\mathrm{total}\;\mathrm{of}\;28.)\;(M_{s}=1$)
$\mathrm{Damping}\;\mathrm{ratio}\;\mathrm{of}\;\mathrm{main}\;\mathrm{structure}\;(\xi_{s}$)	1, 2, 3, 5, 7.5, 10% (A total of 6.)
$\mathrm{Damping}\;\mathrm{ratio}\;\mathrm{of}\;\mathrm{TMD}\;(\xi_{d}$)	$0.01\;\leq \;\xi_{d}$ ≤ 0.3
$\mathrm{Period}\;\mathrm{of}\;\mathrm{TMD}\;(T_{d}$)	$0.8T_{s}$$\;\leq \;T_{d}$$\;\leq \;1.5T_{s}$$\;(T_{s}=1$)

**Table 5:** Parameter results of Explicit Formula 1.

Parameter	a ($\mathrm{or}\;a^{\prime }$)	b ($\mathrm{or}\;b^{\prime }$)	c ($\mathrm{or}\;c^{\prime }$)	d ($\mathrm{or}\;d^{\prime }$)	$R^{2}$
$\xi_{d,opt}$	0.0031	9.7353	0.2484	0.6354	0.9999
$f_{opt}$	1.2263	3.8063	1.0708	5.2784	0.9964

**Table 6:** Parameter results of Explicit formula 2.

Parameter	a ($\mathrm{or}\;a^{\prime }$)	b ($\mathrm{or}\;b^{\prime }$)	$R^{2}$
$\xi_{d,opt}$	0.2494	0.6679	0.9999
$f_{opt}$	1.2263	7.0426	0.9961

**Table 7:** Characteristics of MDOF structure.

Story	Mass (ton)	Stiffness (kN/m)	Damping (kN s/m)	First Mode Shape
10	98	34,310	442.599	1.3590
9	107	37,430	482.847	1.3211
8	116	40,550	523.095	1.2480
7	125	43,670	536.343	1.1460
6	134	46,790	603.591	1.0190
5	143	49,910	643.839	0.8710
4	152	53,020	683.958	0.7080
3	161	56,140	724.206	0.5340
2	170	52,260	674.154	0.3550
1	179	62,470	805.863	0.1750

**Table 8:** Optimal results for 10-story structure with TMD and structural responses (μ = 0.05) from explicit formulas.

	Without TMD	With TMD	With TMD	With TMD	With TMD	With TMD	With TMD
		Sadek et al. [22]	Bakre et al. [21]	Leung et al. [9]	Salvi et al. [24]	Explicit Formula 1	Explicit Formula 2
$\xi_{d,opt}(\%)$		32.24	14.92	14.91	15.19	14.94	14.95
$f_{opt}(w_{d}/w_{s})$		0.9316	0.9078	0.9088	0.9660	0.9068	0.9053
$m_{d}(ton)$		55.450	55.450	55.450	55.450	55.450	55.450
$c_{d}(kNs/m)$		103.615	46.725	46.731	50.625	46.711	46.671
$k_{d}(kN/m)$		465.561	442.023	442.983	500.528	441.027	439.560
$u_{1}(m)$	0.0410	0.0348	0.0329	0.0329	0.0341	0.0328	0.0328
$u_{10}(m)$	0.3270	0.2735	0.2688	0.2690	0.2800	0.2686	0.2682
$H_{2}\;norm$	1.9618	1.0698	0.9679	0.9680	0.9964	0.9679	0.9678

**Table 9:** Optimal results for 10-story structure with TMD and structural responses (μ = 0.05) using Explicit formulas 1 and 2 and previous studies.

	Without TMD	With TMD	With TMD	With TMD	With TMD	With TMD	With TMD	With TMD
		Warburton [7]	Sadek et al. [22]	Hadi et al. [8]	Kaveh et al. [28]	Etedali et al. [15]	Explicit Formula 1	Explicit Formula 2
$\xi_{d,opt}(\%)$		14.76	32.54	15.37	10.76	6.96	14.94	14.95
$f_{opt}(w_{d}/w_{s})$		0.8940	0.9302	0.9035	0.8144	0.8393	0.9068	0.9053
$m_{d}(ton)$		55.450	55.450	55.450	55.450	55.450	55.450	55.450
$c_{d}(kNs/m)$		45.500	104.400	47.900	30.234	20.144	46.711	46.671
$k_{d}(kN/m)$		428.700	464.100	437.900	355.758	377.800	441.027	439.560
$u_{1}(m)$	0.0410	0.0360	0.0360	0.0340	0.0306	0.0300	0.0328	0.0328
$u_{10}(m)$	0.3270	0.3100	0.2810	0.2720	0.2427	0.2430	0.2686	0.2682
$H_{2}\;norm$	1.9618	0.9688	1.0714	0.9677	1.0653	1.1184	0.9679	0.9678

## Data Availability

Data are contained within the article. Data from this research can be accessed upon request by contacting the first author.

## Appendix A

**Table A1:** Optimal TMD parameter for a damped SDOF structure based on GA for specified mass ratio.

μ	$\xi_{s}=0.01$		$\xi_{s}=0.02$		$\xi_{s}=0.03$		$\xi_{s}=0.05$		$\xi_{s}=0.075$		$\xi_{s}=0.01$	
	$\xi_{d,opt}$	$f_{opt}$	$\xi_{d,opt}$	$f_{opt}$	$\xi_{d,opt}$	$f_{opt}$	$\xi_{d,opt}$	$f_{opt}$	$\xi_{d,opt}$	$f_{opt}$	$\xi_{d,opt}$	$f_{opt}$
0.001	0.016	0.998	0.016	0.996	0.016	0.994	0.016	0.990	0.016	0.981	0.016	0.971
0.002	0.022	0.996	0.022	0.995	0.022	0.992	0.022	0.987	0.022	0.978	0.022	0.967
0.003	0.027	0.995	0.027	0.993	0.027	0.990	0.027	0.984	0.027	0.975	0.027	0.963
0.004	0.032	0.993	0.031	0.991	0.032	0.988	0.032	0.982	0.032	0.972	0.031	0.960
0.005	0.035	0.992	0.035	0.989	0.035	0.987	0.035	0.980	0.035	0.969	0.035	0.957
0.006	0.039	0.990	0.039	0.988	0.039	0.985	0.039	0.978	0.039	0.967	0.038	0.954
0.007	0.042	0.989	0.042	0.986	0.042	0.983	0.042	0.976	0.042	0.965	0.042	0.951
0.008	0.045	0.987	0.044	0.985	0.045	0.982	0.045	0.974	0.044	0.962	0.045	0.948
0.009	0.047	0.986	0.047	0.983	0.047	0.980	0.047	0.972	0.047	0.960	0.047	0.946
0.01	0.050	0.985	0.050	0.982	0.050	0.978	0.050	0.970	0.050	0.958	0.050	0.944
0.015	0.061	0.978	0.061	0.975	0.061	0.971	0.061	0.962	0.061	0.948	0.061	0.933
0.02	0.070	0.972	0.070	0.968	0.070	0.963	0.070	0.953	0.070	0.939	0.070	0.922
0.025	0.078	0.966	0.078	0.961	0.078	0.956	0.078	0.946	0.078	0.930	0.078	0.912
0.03	0.086	0.959	0.086	0.954	0.086	0.950	0.086	0.938	0.086	0.922	0.086	0.903
0.035	0.093	0.953	0.093	0.948	0.092	0.943	0.092	0.931	0.092	0.913	0.092	0.894
0.04	0.098	0.947	0.099	0.942	0.099	0.936	0.099	0.923	0.099	0.906	0.099	0.886
0.045	0.104	0.941	0.104	0.936	0.105	0.930	0.104	0.916	0.105	0.898	0.104	0.878
0.05	0.110	0.935	0.110	0.929	0.110	0.923	0.110	0.910	0.110	0.891	0.110	0.870
0.055	0.115	0.929	0.115	0.923	0.115	0.917	0.115	0.903	0.115	0.884	0.115	0.862
0.06	0.120	0.923	0.120	0.917	0.120	0.910	0.120	0.896	0.120	0.876	0.120	0.854
0.065	0.124	0.917	0.125	0.911	0.125	0.904	0.125	0.890	0.125	0.870	0.124	0.847
0.07	0.129	0.912	0.129	0.905	0.129	0.898	0.129	0.883	0.129	0.863	0.129	0.840
0.075	0.133	0.906	0.133	0.899	0.133	0.892	0.133	0.877	0.133	0.856	0.134	0.833
0.08	0.138	0.901	0.138	0.894	0.138	0.886	0.138	0.871	0.138	0.849	0.138	0.826
0.085	0.142	0.895	0.142	0.888	0.142	0.881	0.142	0.865	0.141	0.843	0.142	0.819
0.09	0.145	0.890	0.145	0.882	0.145	0.875	0.146	0.859	0.146	0.836	0.146	0.812
0.095	0.149	0.884	0.149	0.877	0.149	0.869	0.149	0.853	0.149	0.830	0.149	0.805
0.1	0.153	0.879	0.153	0.871	0.153	0.863	0.153	0.847	0.153	0.824	0.153	0.799
